# Cardiovascular Pharmacotherapy and Falls in Old People: Risks and Prevention—An Observational Case–Control Study

**DOI:** 10.3390/jcm14134570

**Published:** 2025-06-27

**Authors:** Sorina Maria Aurelian, Anca Iuliana Pîslaru, Sabinne-Marie Albișteanu, Sonia Dragoescu, Sandra Monica Gîdei, Adina Carmen Ilie, Ramona Ștefăniu, Corina Oancea, Ana-Gabriela Prada, Ioana Dana Alexa

**Affiliations:** 1 Clinic of Geriatrics, Hospital of Chronic Diseases “Sf. Luca”, Faculty of Medicine, University of Medicine and Pharmacy “Carol Davila”, 041915 Bucharest, Romania; sorina.aurelian@umfcd.ro (S.M.A.); sandra-monica.gidei@drd.umfcd.ro (S.M.G.); ana-gabriela.prada@drd.umfcd.ro (A.-G.P.); 2 Department of Physical Medicine and Rehabilitation, Faculty of Medicine, University of Medicine and Pharmacy “Carol Davila”, 050474 Bucharest, Romania; corina.oancea@umfcd.ro; 3 Department of Medical Specialties II, Faculty of Medicine, University of Medicine and Pharmacy “Grigore T Popa”, 700115 Iasi, Romania; sabinne-marie.taranu@umfiasi.ro (S.-M.A.); adina.ilie@umfiasi.ro (A.C.I.); ramona.stefaniu@umfiasi.ro (R.Ș.); ioana.alexa@umfiasi.ro (I.D.A.); 4 Clinic of Internal Medicine, Emergency Teaching Hospital “Sf. Ioan”, Faculty of Medicine, University of Medicine and Pharmacy “Carol Davila”, 041915 Bucharest, Romania; sdragoesc@gmail.com

**Keywords:** fall risk, cardiovascular medication, older adults

## Abstract

**Background:** Falls are a major cause of morbidity and mortality among older adults and are influenced by comorbidities and polypharmacy. Cardiovascular diseases (CVDs) and their associated treatments are particularly prevalent in this population and may contribute to fall risk. **Objectives:** The objectives of this study were to examine the association between cardiovascular pharmacotherapy and fall risk in older adults and to identify potential preventive strategies. **Methods:** This observational case–control study was conducted between June and December 2024 and included 200 participants aged over 55 years who provided informed consent. Participants were assessed using the Downton Fall Risk Index and divided into two equal groups, with those at high risk of falling and controls. All participants underwent a comprehensive geriatric assessment, including anamnesis, clinical evaluation, and laboratory testing focused on cardiovascular risk factors. The prevalence of CVD and the use of specific cardiovascular medications were analyzed. **Results:** Patients at high risk of falling showed significant differences compared to the control group in several parameters, including systolic blood pressure (SBP: 140.41 mmHg vs. 151.28 mmHg, *p* = 0.001), ankle brachial index (left ABI: 1.09 vs. 1.15., *p* = 0.033), and presence of cardiovascular diseases (*p* = 0.001), as well as total cholesterol (*p* = 0.005) and triglyceride levels (*p* = 0.047). Certain cardiovascular medications were significantly associated with increased fall risk, including spironolactone (OR = 4.10, *p* = 0.001), beta-blockers (OR = 1.88, *p* = 0.031), and calcium channel blockers (OR = 2.05, *p* = 0.014), especially in combination with one another. Additional risk factors included frailty, cognitive impairment, diabetes, and neurological or osteoarticular conditions. Interventions such as medication review, deprescribing, and dosage adjustments may help reduce fall risk without compromising cardiovascular disease management. **Conclusions:** Cardiovascular diseases and related pharmacotherapy are significantly associated with an increased risk of falls in older adults. Regular medication reviews, deprescribing where appropriate, and individualized treatment plans may help minimize fall risk while maintaining the effective cardiovascular care of this vulnerable population.

## 1. Introduction

With the accelerating aging of the population, the health of older adults is becoming a major global concern. It is estimated that by the year 2050, the proportion of older individuals in Europe will quadruple compared to 2010, largely due to advances in early diagnostic techniques and effective treatments that contribute to increased life expectancy and reduced mortality [1]. Cardiovascular disease remains the most prevalent pathology, affecting approximately 40% of individuals aged 40 to 59 and up to 75% of those aged 60 to 79. The highest reported prevalence is after the age of 80 [2]. Ischemic heart disease dominates the pathological landscape, serving as a major cause of morbidity, disability, and mortality among older adults. It requires complex pharmacological strategies, often in the face of multimorbidity driving radical therapeutic decisions that may amplify iatrogenic risks. Aging-associated conditions, such as frailty, dementia, osteoarthritis, and diabetes mellitus exacerbate cardiovascular disease progression and complicate diagnosis, prognosis, and treatment [3,4].

Older adults with cardiovascular diseases face an increased risk of falling. About one-third of individuals over 65 experience at least one fall annually, often with severe consequences [5,6]. Falls impact functional independence, accelerate physical and cognitive decline, and are linked to depression, anxiety, social isolation, and increased use of medical services. Additionally, the trauma-derived fear of falling again, which is particularly prevalent among older patients who have experienced a fall, leads to reduced physical activity, worsening frailty, and further functional decline [5,6]. Fall risk is multifactorial, involving intrinsic and extrinsic factors such as cardiovascular disease (including acute events), decompensated osteoarticular pathology, and polypharmacy. In particular, cardiovascular medication side effects might include orthostatic hypotension, bradycardia, or vertigo [6,7]. Polypharmacy, defined as the simultaneous use of five or more medications, is increasingly common, affecting between 20% and 60% of older adults. It increases adverse drug reactions, hospitalizations, and functional decline. As individuals age, physiological changes, such as the decline in hepatic and renal function, increase the body’s sensitivity to the adverse effects of pharmacotherapy. Many cardiovascular agents (statins, antihypertensives, anticoagulants) that dominate the therapeutic regimen of older adults are associated with increased fall risk. Therapeutic adherence, dependent on cognitive, psycho-emotional, and autonomy status, is crucial. Thus, comprehensive geriatric assessment (CGA) is key in managing older patients [7,8,9,10].

Fall prevention is critically important. Although interventions are effective, accurately identifying at-risk individuals remains challenging. Prognostic models integrating multiple risk factors may help stratify risk and personalize prevention. Deprescribing and medication review are important but should be part of a broader multifactorial strategy [11]. Each older adult’s unique phenotype and resilience merit personalized therapeutic approaches, including risk stratification and dosage adjustment. There remains a need for studies clarifying which cardiovascular medications most contribute to fall risk and how this risk is influenced by geriatric syndromes.

This study hypothesizes that cardiovascular pharmacotherapy—particularly in the context of polypharmacy and geriatric syndromes—increases the risk of falling in older adults. The topic was chosen due to the clinical challenges posed by pervasive cardiovascular disease in seniors, especially with regard to safe pharmacological management. The main objective of the study is to assess the association between cardiovascular treatments and fall risk and to identify practical individualized preventive strategies. Specifically, the study aims to evaluate the impact of cardiovascular medications on fall incidence and to propose safer clinical approaches tailored to the needs of older patients.

## 2. Materials and Methods

### 2.1. Study Design

In this case–control study, we analyzed a group of 200 patients admitted to the geriatrics department of “Sf. Luca” Chronic Disease Hospital, Bucharest, Romania, between June and December 2024. The study protocol was approved by the Ethics Committee of the hospital under approval number [371/2025], and all procedures complied with the principles outlined in the Declaration of Helsinki. All enrolled patients were hospitalized for chronic cardiovascular disease. They gave written and oral informed consent to participate in the study. To reduce selection bias, all potentially eligible patients admitted to the geriatrics department during the study period were screened for inclusion.

Generative artificial intelligence (GenAI) was not used in this study or in the writing of this paper.

#### 2.1.1. Inclusion Criteria

Eligible patients were men and women aged 55 and older with cardiovascular pathology who had been prescribed associated medication and who provided written, informed consent. All participants had no severe comorbidities in order to limit confounding factors.

#### 2.1.2. Exclusion Criteria

Patients with a history of severe comorbidities, such as diabetes complicated by polyneuropathy, severe neurological disorders, or advanced osteoarticular disease, were excluded in order to minimize measurement bias, confounding variables, and distortion of Downton scale results.

These exclusion criteria were intended to optimize comparability between cases and controls.

A total of 200 participants were enrolled and evaluated at baseline. All participants met the inclusion criteria, and none were excluded or lost to follow-up. The final analysis included all 200 originally enrolled subjects.

#### 2.1.3. Groups

A comprehensive geriatric assessment (CGA) was performed for all enrolled patients by applying standardized, internationally recognized questionnaires to assess cognitive status. It evaluated mental status (mini-mental status examination, MMSE), emotional status (15-question Geriatric Depression Scale, GDS-15), functional dependence in both basic and instrumental activities of daily living (ADL and IADL tests), and fall risk (Downton Fall Risk Index).

Based on these scales, cognitive impairment was defined as MMSE scores < 25 points, altered psycho-emotional status as GDS-15 scores > 5 points, impaired basic and instrumental autonomy as ADL/IADL scores < 3 points, and increased fall risk as Downton scores > 2 points.

Patients were divided into two groups based on the results of the Downton fall risk index. The high-risk group was represented by a score greater than or equal to 3 points, while the remaining patients were assigned to the control group or low-risk group.

The Downton scale was chosen because it has been validated in Romania, is widely used in most public hospitals across the country, and has been authorized by the Romanian hospital accreditation agency ANMCS (National Authority for Quality Management in Health). The Downton scale tallies objective criteria such as prior falls, chronic medications (sedatives, diuretics, non-diuretic antihypertensives, antiparkinsonian drugs, antidepressants, and other medications), sensory deficits (visual, auditory, or peripheral), mental state, and gait (steady, steady with aid, unsteady with and without a walking aid) [12].

Participants underwent anamnesis and clinical examination to identify cardiovascular diseases and related pharmacotherapy. Results were analyzed based on demographic characteristics and biological parameters. The sample size was constrained by the limited research period (six months) and the number of patients with less complex comorbidities.

### 2.2. Statistical Analysis

Descriptive and analytical statistics were used in the analysis. Information collected from patient records was systematized, coded, and synthesized to generate a database in IBM SPSS version 18.0. The initial processing allowed for the calculation of primary indicators (absolute values), while statistical comparison and generalization methods were used to compute derived indicators, highlighting qualitative aspects and interdependencies between variables.

Measures of central tendency (mean, median, mode, minimum and maximum values) and measures of dispersion (standard deviation, coefficient of variation) were applied. The skewness test (−2 < *p* < 2) confirmed the homogeneity of the data. Quantitative variables were handled using appropriate statistical methods, and variable groupings were based on clinical cutoffs (e.g., MMSE < 25).

The univariate analysis sought significant differences between groups at a significance level of 95%. For quantitative variables, the *t*-Student test was applied, while for qualitative variables, the chi-squared test and the Kruskal–Wallis tests were used. Additionally, the ROC curve was employed to evaluate the predictability of dependent variables based on independent variables, where a larger area under the curve indicates higher predictability.

No missing data were recorded during data collection, so no imputation or correction methods were required.

## 3. Results

### 3.1. Participants and Data Completeness

A total of 200 participants were enrolled and included in the analysis. All eligible individuals were assessed at baseline, with no exclusions or patients lost to follow-up during the study. Therefore, a flow diagram was considered unnecessary. Reasons for non-participation at each stage were not applicable, as all eligible participants were included.

### 3.2. Descriptive and Outcome Data

Baseline characteristics of the study participants, including demographic, clinical, and social factors, are presented in Table 1, Table 2 and Table 3. There was no missing data for the main variables of interest. Outcome data and summary measures of exposure are presented by study group in Table 1, Table 2, Table 3, Table 4, Table 5 and Table 6.

### 3.3. Statistical Analysis and Main Results

All statistical analyses were unadjusted; no multivariate adjustments for confounders were performed. The cut-off values for categorizing continuous variables (e.g., age ≥ 75 years) are specified in the Methods Section. Unadjusted estimates, including *p*-values and 95% confidence intervals where appropriate, are reported in the results tables. The translation of relative risk estimates into absolute risk over a specific time period was not performed due to the cross-sectional study design.

Cohorts were homogeneous in terms of gender, rural/urban origin, and educational level. In both groups, female participants predominated (80% vs. 77%; *p* = 0.365), with a higher proportion from urban areas (71% vs. 77%; *p* = 0.210) and a lower educational level (vocational or secondary education or less) (54% vs. 63%; *p* = 0.474) (Table 1).

In the high-fall-risk group, 69% of patients were over the age of 75, while in the non-risk group, this proportion was 40% (*p* = 0.001) (Table 1). The frequency of smokers was low in both study cohorts (14% vs. 16%; *p* = 0.282).

Among the clinical parameters monitored, the group with high fall risk showed significantly lower mean values for systolic blood pressure (SBP) (140.41 vs. 151.28 mmHg; *p* = 0.001), diastolic blood pressure (DBP) (78.55 vs. 82.40 mmHg; *p* = 0.015), and ankle brachial index (ABI) (1.09 vs. 1.15; *p* = 0.033). The average number of cardiovascular diseases and the number of cardiovascular drugs were significantly higher in the high-fall-risk group (*p* = 0.001, *p* = 0.007) (Table 2).

**Figure 1:**
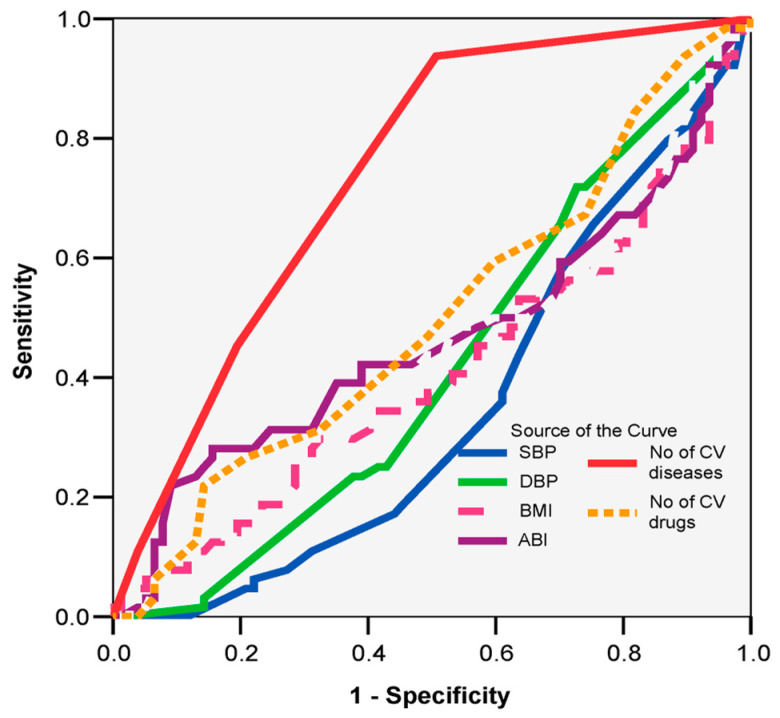
ROC curve: prognostic clinical parameters of increased fall risk.

Among the clinical parameters, only the number of cardiovascular diseases is confirmed as a good predictor of increased fall risk (AUC = 0.741; 95% CI: 0.659–0.822; *p* = 0.001), with more than three cardiovascular diseases showing a sensitivity of 93% and a specificity of 49% (Figure 1).

Among the biological parameters monitored (Table 3), only the mean values of total cholesterol (198.92 vs. 178.36; *p* = 0.005) and triglycerides (134.74 vs. 112.70; *p* = 0.047) were significantly higher in the group with increased fall risk.

A univariate analysis of the monitored geriatric assessment scores highlighted the following significant differences based on fall risk (Table 4).

For patients with high fall risk:

- The ADL score classified 54% of patients as independent (*p* = 0.003).
- The IADL score classified 39% of patients as independent (*p* = 0.002).
- The MMSE score classified 67% of patients as having normal values and 21% as having mild cognitive impairment (*p* = 0.009).

No significant percentage differences in comorbidities were recorded based on fall risk (*p* > 0.05) (Table 5).

In the high-fall-risk group, there were four times as many patients receiving spironolactone than in the control group (OR = 4.10; 95% CI: 2.05–8.18; *p* = 0.001). Approximately twice as many received beta-blockers (OR = 1.88; 95% CI: 1.01–3.50; *p* = 0.031) and calcium channel blockers (OR = 2.05; 95% CI: 1.15–3.66; *p* = 0.014) approximately twice as often compared to patients without a high risk of falling (Table 6).

### 3.4. Additional Analyses

No subgroup or sensitivity analyses were conducted.

## 4. Discussion

In our study, we observed that patients with a high risk of falling had significantly lower mean systolic (SBP) and diastolic blood pressure (DBP) values compared to the control group. These findings reinforce the likelihood of a causal relationship between hypotension and increased fall risk in older adults.

Paraclinical evaluation showed that the high-risk group had lower ankle brachial index (ABI) values, implicating peripheral arterial disease (PAD) as a predisposing factor. Considering the aging population and rising cardiovascular disease prevalence, assessing fall risk when prescribing cardiovascular medications is increasingly important, as these treatments can impact balance, cognition, and autonomy [2,5,7].

In our cohort, being over 75 was significantly associated with a higher fall risk, consistent with the literature highlighting age, frailty, female sex, and prior falls as strong predictors [13].

A more recent detailed meta-analysis extrapolates predisposing factors for fall risk. It identifies advanced age, frailty, malnutrition, alcohol and tobacco use, polypharmacy, and chronic diseases (such as heart disease, hypertension, diabetes, stroke, and Parkinson’s disease) as strongly associated with an increased risk of falls in the elderly. The study emphasizes the benefit of evaluating multiple aspects of lifestyle, comorbidities, and current medication in the prevention of falls among older adults. Furthermore, advanced age may serve as a screening criterion [14].

One analysis found orthostatic hypotension measured after 4.5 min of standing more predictive of falls than early measurements [15]. These findings strengthen our conclusions, highlighting the value of comprehensive blood pressure assessment in elderly patients. In addition, our clinical experience in both inpatient care and outpatient follow-up is consistent with these findings. Consequently, by performing regular detailed blood pressure measurements, as in the Schellong test, the prognostic value and therapeutic effectiveness of this method can be confirmed, particularly in patients for whom efforts are being made to reduce polypharmacy through the deprescribing of excessive antihypertensive medication [16,17].

An increasing number of studies recommend evaluating orthostatic blood pressure and deprescribing fall-risk-increasing drugs, especially since hypotension is a common adverse effect of many necessary medications [18,19,20,21]. Peripheral artery disease is also a recognized fall risk factor, affecting muscle function, gait, and balance, and a growing body of research aims to refine fall risk tests for this group [22,23].

In the context of peripheral artery disease, polypharmacy, particularly involving cardiovascular medications, represents a significant risk factor for falls in older patients. A recently published study demonstrated that the revascularization of patients with peripheral arterial disease had an increased risk of cardiovascular death, major adverse events, a sustained risk of falling, and a higher likelihood of requiring reintervention when on multiple medications. These findings align with our results and underscore the importance of a balanced approach in managing pharmacotherapy in patients with peripheral arterial disease to reduce fall risk and improve clinical outcomes [24].

According to our findings, comorbidities considered individually or independently do not significantly influence fall risk, as seen in the literature [25,26]. However, the average number of cardiovascular diseases was significantly higher in the high-fall-risk group.

Our results demonstrate a statistically significant association between elevated levels of triglycerides and total cholesterol and an increased risk of falls. These findings suggest a possible link between lipid profiles and physical frailty—an association also reported in the scientific literature, where dyslipidemia has been correlated with systemic inflammation and impaired neuromuscular function, both of which are factors in the pathogenesis of falls in older people [27,28,29].

The results of the univariate analysis in our study provoke several interesting insights into functional and cognitive assessment scores and fall risk in older patients. According to the ADL score, 54% of patients with a high fall risk are still functionally independent. This finding, although counterintuitive, can be explained by how the loss of independence in basic activities is not always an early predictor of falls. Falls can occur even in seemingly robust individuals and may be driven by other factors, such as polypharmacy, orthostatic instability, or subtle impairments of motor coordination [30,31,32].

In contrast, the IADL score, which assesses more complex tasks, indicated a lower degree of independence (39%) among the same patients, a finding confirmed by the literature. This suggests that a decline in complex activities is an early marker of frailty and, implicitly, of fall risk. Thus, IADL was shown to be a more sensitive indicator than ADL in detecting fall risk in this population [33].

Additionally, the MMSE score revealed that, although most patients in the study had intact cognitive function, a significant proportion (21%) displayed mild cognitive impairment. Both categories exhibited an increased risk of falls. The explanation for this correlation in this vulnerable, older population points to deficits in executive functions and processing speed; gait and balance disturbances; reduced perception of risk; and unsafe behaviors. The effects of polypharmacy and iatrogenic factors contribute to the exacerbation of these effects and, implicitly, to an increased fall risk. The recent literature increasingly provides supportive data in this regard [34,35,36].

Therefore, periodic medication review is essential for identifying and eliminating medications that may impair cognitive function or physical balance in older adults. The Beers Criteria, updated by the American Geriatrics Society in 2023, provide a list of potentially inappropriate medications for older adults, including some antihypertensives, antidepressants, benzodiazepines, and antipsychotics associated with an increased risk of falls [37].

The results of our study indicate a significant association between the use of certain cardiovascular medications and an increased risk of falls in older patients. In particular, the use of spironolactone was more than four times more frequent among patients with a high fall risk. This observation aligns with the recent literature, which highlights that diuretics, particularly potassium-sparing ones, and sodium–glucose co-transporter 2 inhibitors (SGLT2is), can contribute to electrolyte imbalances and orthostatic hypotension, factors known to increase the risk of falls in older adults [10,38,39].

A recent study confirms our findings and implicates calcium channel blockers, beta-blockers, and diuretics in increasing the risk of falls in patients with heart failure. This is seen both upon hospital admission and at discharge and with modified doses and volume depletion [40].

These key results support our initial objective of identifying cardiovascular and functional predictors of fall risk in elderly patients, highlighting the complex interplay between medication use, comorbidities, and physiological markers.

The 2024 ESC guidelines stipulate that the prescription of β-blockers for hypertension in the elderly should be restricted to cases with explicit clinical rationale due to increasing evidence of their diminished efficacy and possible harmful consequences in this demographic.

The guidelines underscore the judicious application of β-blockers—specifically β1-selective medicines such as bisoprolol or metoprolol—in senior patients with significant cardiovascular comorbidities, emphasizing their limited utility in the management of isolated hypertension [41].

Indeed, in the context of the risks associated with cardiovascular pharmacotherapy in older adults, fall prevention strategies must be multidimensional, personalized, and based on a comprehensive assessment of risk factors [42,43,44]. Tailoring interventions to each patient’s functional and medical profile is crucial to the effectiveness of fall prevention programs [45,46,47,48,49,50].

In the hospital setting, fall prevention among older patients treated with cardiovascular medication requires an integrated approach that combines multifactorial assessment, specialist-guided exercises based on functional status, and occupational and psychological therapies. The literature also highlights interventions addressing vestibular dysfunction, pain, and impairment of vision and hearing. Exercise programs should be continued post-discharge, with support from telemedicine and assistive technologies to prevent functional regression. The integration of these strategies into a comprehensive care plan can significantly reduce the incidence of falls and associated complications in this population [51].

The main limitations of the study are related to its small sample size and the inability to establish causal relationships between factors, given the case–control design of the research. Selection bias is possible despite efforts to minimize it, as patients were recruited from a single center. Future studies with a more robust design, such as longitudinal research, would be useful for evaluating causal relationships.

Our findings highlight the importance of interdisciplinary personalized care in senior patients with cardiovascular comorbidities. A regular assessment of functional and cognitive status, blood pressure, ankle brachial index, and lipid profiles—alongside medication optimization—can improve fall prevention. The integration of geriatrics, cardiology, pharmacology, and rehabilitation is essential for improving outcomes and quality of life in this vulnerable population, emphasizing the multidisciplinary needs of the frail senior patient.

These results may be generalized to similar patient groups—senior patients with cardiovascular disease, polypharmacy, and functional decline—especially in hospital settings. The consistency with the existing literature supports broader applicability in comparable geriatric populations.

## 5. Conclusions

The study emphasizes the importance of identifying the risk factors associated with falls among the elderly, especially in the context of cardiovascular pharmacotherapy. The results confirm that advanced age, polypharmacy, arterial hypotension, cardiovascular diseases, and dyslipidemia are significant factors contributing to the increased risk of falls in older patients. The early identification and management of cardiovascular risks and polypharmacy play a crucial role in preventing falls in older adults. Targeted deprescribing strategies, along with regular assessments of blood pressure profiles, as well as cognitive and motor functions, could significantly contribute to reducing the risk of falls in this vulnerable group.

Fall prevention strategies must be integrated in a multidimensional approach that includes lifestyle modifications, functional and cognitive assessment, and the reduction in iatrogenic effects through individualized deprescribing. Comprehensive geriatric assessment (particularly cognitive executive function and autonomy level), paraclinical monitoring (blood pressure fluctuations, ankle brachial index), and biological assessment (lipid profile) are essential in identifying whether patients are at a high risk for falls. Clinicians must tailor cardiovascular drug therapy to each patient’s comorbidities, drug interactions, and fall risk. Medications commonly prescribed to older patients, such as beta-blockers, potassium-sparing diuretics, or calcium channel blockers, should be carefully adjusted or deprescribed to balance cardiovascular benefits and fall risk. Therefore, future research should focus on larger, multicenter longitudinal studies to better assess causal relationships between cardiovascular risk factors, pharmacotherapy, and fall risk in older adults.

## Figures and Tables

**Table 1:** Socio-demographic characteristics of the study group.

Characteristics	Total Group (*n* = 200)		Low-Risk Group (*n* = 100)		High-Risk Group (*n* = 100)		Chi-Squared Test *p*
	*n*	%	*n*	%	*n*	%	
**Sex**							0.365
**Male**	43	21.5	20	20.0	23	23.0	
**Female**	157	78.5	80	80.0	77	77.0	
**Age**							**0.001**
**<75 years**	91	45.5	60	60.0	31	31.0	
**≥75 years**	109	54.5	40	40.0	69	69.0	
**Place of Residence**							0.210
**Urban**	148	74.0	71	71.0	77	77.0	
**Rural**	52	26.0	29	29.0	23	23.0	
**Educational Level**							0.474
**None**	68	34.0	29	29.0	39	39.0	
**Primary**	10	5.0	6	6.0	4	4.0	
**Secondary**	30	15.0	16	16.0	14	14.0	
**Vocational school**	9	4.5	3	3.0	6	6.0	
**High school**	33	16.5	16	16.0	17	17.0	
**Post-secondary**	28	14.0	18	18.0	10	10.0	
**University**	22	11.0	12	12.0	10	10.0	
**Smoker**							0.282
**Yes**	32	16.0	14	14.0	18	16.0	
**No**	168	84.0	86	86.0	82	77.0	

**Table 2:** Comparative clinical parameters by study group.

Parameter	Low-Risk Group (*n* = 100)	High-Risk Group (*n* = 100)	*t*-Student Test
Systolic Blood Pressure	151.28 ± 27.98	140.41 ± 18.58	**0.001**
Diastolic Blood Pressure	82.40 ± 11.51	78.55 ± 10.28	**0.015**
Body Mass Index	30.32 ± 5.41	30.11 ± 7.53	0.828
Ankle Brachial Index	1.15 ± 0.16	1.09 ± 0.20	**0.033**
Number of Cardiovascular Diseases	2 ± 1	3 ± 1	**0.001**
Number of Cardiovascular Drugs	3 ± 1	4 ± 1	**0.007**

**Table 3:** Comparative biomarkers in study groups.

Biomarker	Low-Risk Group (*n* = 100)	High-Risk Group (*n* = 100)	*t*-Student Test
Uric acid	5.44 ± 1.38	5.62 ± 5.60	0.757
Glucose	115.16 ± 45.82	110.20 ± 28.80	0.363
HbA1c	6.45 ± 1.13	6.09 ± 1.27	0.193
Total cholesterol	178.36 ± 48.25	198.92 ± 53.26	**0.005**
LDL cholesterol	114.65 ± 43.88	127.36 ± 75.17	0.146
HDL cholesterol	53.64 ± 15.98	56.86 ± 16.46	0.163
Triglycerides	112.70 ± 60.59	134.74 ± 92.23	**0.047**

**Table 4:** Geriatric assessment scores in the study group.

Scores	Total Group (*n* = 200)		Low-Risk Group (*n* = 100)		High-Risk Group (*n* = 100)		Chi-Squared Test *p*
	*n*	%	*n*	%	*n*	%	
**ADL**							
**Independent**	128	64.0	74	74.0	54	54.0	**0.003**
**Partially dependent**	55	27.5	20	20.0	35	35.0	0.288
**Totally dependent**	17	8.5	6	6.0	11	11.0	0.206
**IADL**							
**Independent**	100	50.0	61	61.0	39	39.0	**0.002**
**Partially dependent**	49	24.5	19	19.0	30	30.0	0.071
**Totally dependent**	51	25.5	20	20.0	31	31.0	0.075
**GDS**							
**Normal**	116	58.0	60	60.0	56	56.0	0.568
**Mildly depressed**	66	33.0	29	29.0	37	37.0	0.230
**Very depressed**	18	9.0	11	11.0	7	7.0	0.324
**MMSE**							
**Normal**	150	75.0	83	83.0	67	67.0	**0.009**
**Mild impairment**	29	14.5	8	8.0	21	21.0	**0.009**
**Moderate impairment**	16	8.0	6	6.0	10	10.0	0.298
**Severe impairment**	5	2.5	3	3.0	2	2.0	0.651

**Table 5:** Estimated fall risk in the presence of personal medical history.

Medical History	High-Risk Group (*n* = 100)		Low-Risk Group (*n* = 100)		Chi2 Test	Estimated Risk	IC95%
	*n*	%	*n*	%			
Hypertension	91	91.0	95	95.0	0.129	0.71	0.48–1.05
Arrhythmia	33	33.0	28	28.0	0.270	1.12	0.84–1.50
Valvulopathy	20	20.0	17	17.0	0.358	1.10	0.79–1.54
Cerebrovascular incidents (BCI)	60	60.0	62	62.0	0.442	0.96	0.72–1.27
Heart failure	31	31.0	30	30.0	0.500	1.02	0.76–1.38

**Table 6:** Treatment based on fall risk.

Treatment	High-Risk Group (*n* = 100)		Low-Risk Group (*n* = 100)		Chi-Squared Test	OR	IC95%
	*n*	%	*n*	%			
Beta-blockers	77	77.0	64	64.0	**0.031**	1.88	1.01–3.50
Calcium channel blockers	48	48.0	31	31.0	**0.014**	2.05	1.15–3.66
ACE inhibitors	52	52.0	54	54.0	0.777	0.92	0.53–1.61
Thiazide diuretics	56	56.0	62	62.0	0.390	0.78	0.44–1.37
Spironolactone	40	40.0	14	14.0	**0.001**	4.10	2.05–8.18
Nitrates	15	15.0	12	12.0	0.536	1.29	0.57–2.93
SGLT2 inhibitors	8	8.0	5	5.0	0.391	0.65	0.52–5.24
ARBs	35	35.0	25	25.0	0.124	1.62	0.87–2.98

## Data Availability

The original contributions presented in this study are included in the article. Further inquiries can be directed to the corresponding author.
